# Supplementary material for: Tooth replacement patterns in the Early Triassic epicynodont Galesaurus planiceps (Therapsida, Cynodontia)
Source: PLoS One. 2020 Dec 30;15(12):e0243985. doi: 10.1371/journal.pone.0243985 (PMC7773207; doi:10.1371/journal.pone.0243985)
Supplement: Attachment — Submitted filename: Norton-2020-Galesaurus-review.docx [file pone.0243985.s003.docx]

Review of “Tooth replacement patterns in the Early Triassic epicynodont Galesaurus

planiceps (Therapsida, Cynodontia)” Manuscript for PONEW by Norton, L. A., Abdala, F. Rubidge B. S. and J. Botha

By: Zhe-Xi Luo ([zxluo@uchicago.edu](mailto:zxluo@uchicago.edu))

Review Summary

This is a significant study on the tooth replacement pattern of the cynodont Galesaurus planiceps, with broad implications for the evolution of tooth replacement and skull growth in cynodont evolution. Because this cynodont is a Triassic relative of mammals, its anatomical structure, especially the skull growth and tooth replacement patterns, will be very important for interpreting the evolution from cynodonts to mammals.

The characterization of the tooth replacement is detailed, informative, and excellent. The CT visualization is also very beautiful and very appealing. It adds significant new information on the wider evolutionary variation of the tooth morphology and replacement pattern for epicynodonts (a clade include mammals).

This is also a thorough paper, and well done. I agree with the key conclusions by the authors, and strongly support for Plos-One to publish this paper.

Suggestions for Revision

The paper is publishable – in a very good shape already. I have several suggestions for optional revision. But none of these is about the substance. I offer these comments, in the spirit of making the paper even better, and to make the paper a more useful key reference for other paleontologists, like myself.

Suggestion 1

P2 Lines 33-41 – Abstract

The several sentences of the abstract (lines 33-41) should be re-arranged slightly so the delivery would be more orderly. I suggest these changes of the abstract

How about change to “….. The growth series of Galesaurus shows that the incisors and canine continue to develop and replace, even in the largest (presumably the oldest) specimens of Galesaurus. In Galesaurus, replacement of the canine ceased with the attainment of skeletal maturity, whereas in Thrinaxodon, replacement of the canines continued into adulthood. In the maxilla, the first postcanine is consistently the smallest tooth, showing a proportional reduction in size as skull length increased. The first postcanine locus underwent longer development and sustained its replacement in Galesaurus than in Thrinaxodon. A longer retention of this tooth is a key difference of Galesaurus from Thrinaxodon in which the anterior-most postcanines are lost after replacements. This difference have contributed to the lengthening of the postcanine series as teeth continue to be added to the posterior end of toothrow through ontogeny in Galesaurus, which differs from Thrinaxodon. Overall, there are considerable differences between Galesaurus and Thrinaxodon relating to the replacement and development of their teeth. “

Suggestion 2

The paper will be better if it can add an illustration of CT visualization of the in situ teeth in the mandible in the medial view of the jaw. It is important to illustrate this view, easily obtainable from the CT scans. Several dental replacement features can be shown.

In Thrinaxodon the dental lamina groove (Crompton’s groove) is present on along the margin of the dental alveoli, in the medial view of the mandible. The authors have clearly explained that the tooth alveoli are not ankylosed in Galesaurus, and the gomphodont tooth implant in the alveoli are permanent. By comparison, in Thrinaxodon the deciduous tooth become ankylosed in the dentary (alveoli would be ossified) before the tooth was shed and lost. The dental lamina groove is related to this pattern in Thrinaxodon. Does Galesaurus have the dental lamina groove (for replacement). If dental lamina groove is absent, then there is one more difference from Thrinaxodon.

I suggest that the authors can add this description/discussion with a figure on Galesaurus. This will make the paper better by providing more comparative information to Thrinaxodon.

Suggestion 3

There are now several CT scan studies on Galesaurus skulls (Jasinoski and Abdala 2017; Pusch et al. 2019, and the present manuscript by Norton et al.). But these otherwise excellent papers have not shown a good CT visualization of the postcanine tooth crown. Tooth crowns of Galesaurus are the most unique, among cynodonts , and are quite different from those of Thrinaxodon. Can you illustrated an upper PC crown in medial view, and lateral view. And then do the same for a lower postcanine? The line drawings in Figure 1 are just to simple, and do not show the lingual views.

This will make the paper much more useful, for other paleontologists.

Suggestion 4 (also a neutral comment)

The upper canine can be open-rooted, even in larger and presumably the older skulls (e.g., NMQR135; BP/1/5064). This is most interesting. This could be due to the fact the canine was still slowly growing, or that the tooth is high-crown, or deep rooted, or both.

Interestingly, there is an analogous pattern in the Jurassic haramiyidan Vilevolodon. The replacing lower incisor can also be open-rooted, in the incisor locus that experienced slow tooth growth and replacement in some of these haramiyidans (Luo et al. 2017: extended data figures 4 and 6). I think the open-rooted pattern of incisors in the haramiyidan Vilevolodon may be convergent in the tooth growth mode to the open-rooted and high-crown canines (but obviously these incisor and canine are in different tooth positions).

This is consistent with the authors’ interpretation that Galesaurus has a relatively slow, and sustained tooth replacement.

See: Luo, Z.-X., Meng, Q.-J., Grossnickle, D. M., Liu, D., Zhang, Y.-G., Neander, A. I., and Q, Ji. 2017. New evidence for mammaliaform ear evolution and feeding adaptation in a Jurassic ecosystem. Nature. 548: 326-329 (doi:10.1038/nature23483).
