# Supplementary material for: Tooth replacement patterns in the Early Triassic epicynodont Galesaurus planiceps (Therapsida, Cynodontia)
Source: PLoS One. 2020 Dec 30;15(12):e0243985. doi: 10.1371/journal.pone.0243985 (PMC7773207; doi:10.1371/journal.pone.0243985)
Supplement: Attachment — Submitted filename: Response to Reviewers.docx [file pone.0243985.s004.docx]

**Reviewer 1**

*I have very few substantive reviewer comments for this manuscript. Most of my edits/suggestions are minor organizational issues that I have outlined in the attached PDF.*
—All corrections suggested in the marked-up PDF were incorporated into the manuscript. A detailed list of the changes made are listed below:

- Page 4, Line 82: *Maybe add a brief paragraph on why studying these tooth replacement patterns is important (I'm assuming this would relate to the transition from "reptilian-style" polyphyodonty to oligophyodonty).*—Moved sentence from p. 6 as suggested, and modified to read “Determining the tooth replacement patterns in *Galesaurus* and comparing them to those observed in the previously studied cynodont *Thrinaxodon* [44] may help to ascertain whether any variation in tooth replacement pattern occurred amongst the basal-most cynodonts.”
- Page 6, Lines 117–120: *This should probably be included on page 4 where I included my comment.*—See comment above.
- Page 6, Lines 137–140. *Novelty of study clearly stated.*—No change necessary
- Page 7, Line 148: Inserted “(e.g., tooth replacement)”
- Page 10, Line 200: Inserted “of tooth development”
- Page 12, Line 240: *Do you have a figure number to refer to here?*―Citations to two new figures (Figs 3 and 5) added, as well as a citation to Fig 4 (fig 5 of original submission). The relevant captions were inserted or moved as necessary.
- Page 14, Line 279: Inserted “...and were still in the process of developing”
- Page 14, Line 283: *Move this interpretation of open roots up to the first time you mention open roots in the maxillary incisors.*—Moved “and was still in the process of developing.” Changed “In contrast” to “whereas”
- Page 14, Line 284: *And closed/tapering at the root apex? How do the roots of developing vs. fully developed teeth differ?*—Inserted “…and tapers to a closed point at the root apex” at the end of the sentence.
- Page 15, Lines 308–309: Inserted “is”
- Page 31, Line 798: Inserted “The”
- Page 33, Lines 739–740: *Please reference your graphs here (e.g., Fig. 16).*—Inserted “(e.g., Figs 21 and 22)” and moved captions accordingly. These were figs 16 and 17 of the original submission.
- Page 34, Lines 763–767: *A bit unclear: the previous paragraph and the start of this sentence led me to believe that the PC crowns become more complex in larger individuals, but the end of this statement suggests the opposite. Are you suggesting that the accessory cusps of the PC's are lost through successive replacement events in Galesaurus? Could clarify that a bit here.*—Deleted “This condition is similar to that described for *Diademodon* [49] and *Nanictosaurus* [84], where the distal elements of the postcanine series have a more complex crown morphology. This suggests that the postcanine crown morphologies become more simplified with each successive replacement.” (see also comment on P. 35, L. 806–808)
- Page 35, Line 797: Inserted “the”
- Page 35, Line 798: *So this would be a similar trend to that observed in Galesaurus? Could make a comparison to your findings here.*—Inserted “This condition is similar to that observed in *Galesaurus*.”
- Page 35, Lines 806–808: *This is repetition of the discussion on pg. 34. Please combine these together, or even easier: delete part of the discussion on pg. 34 and leave this one. No need to keep repeating the main findings.*—See comment for P 34, L. 763–767 above. Inserted “and *Nanictosaurus* [84].”
- Page 36, Lines 814–819: *Any inferences to be made about this imbrication? Does it relate to diet in some way? I suggest moving this to the results otherwise.*—This paragraph was moved to the section “Paleobiology of *Galesaurus*”. Inserted “The significance of such an arrangement of the postcanines has yet to be determined, however, in *Galesaurus* the canted orientation of the teeth may have allowed for a greater mesial-distal surface area of each tooth crown to contact the prey during jaw adduction. This may have facilitated the swallowing of proportionally large prey items, relative to the body size of *Galesaurus* [19].”
- Page 36, Line 828: *In Galesaurus or is this a broader statement? If this is meant to be a broader statement, I would add two qualifiers: MARGINAL replacement teeth are always positioned lingual (palatal teeth frequently do not follow this "rule"). And I would also cite Edmund, 1960. If this is a specific reference to Galesaurus, I suggest clarifying that here.*—Inserted “In *Galesaurus*,”
- Page 37, Lines 836–837: *Can you speculate on the direction of the replacement wave? For example, when the odd-numbered teeth are at a different replacement stage, which of them, I1 or I3, is at a more advanced stage?*—Inserted the following paragraph: “It was not possible to determine which direction the replacement wave travelled from the present sample. However, in specimens that show evidence of two or more replacement incisors in a premaxilla (e.g., RC 845, BP/1/5064 and SAM-PK-K10468), the distal teeth of the odd and even-numbered waves tend to be larger than the mesial teeth (i.e., I3 larger than I1, and I4 large than I2). This suggests a back-to-front movement of the replacement wave, but we cannot rule out the idea that the mesial tooth may be smaller, because it represents the initiation of the next replacement wave. If this is correct, then it would support the replacement waves moving in a front-to-back direction.”
- Page 38, Lines 869–873: *Good comparisons here.*—No change necessary
- Page 42, Line 972: Replaced “simple” with “easy”
- Page 43, Line 1007: Inserted “of the entire postcanine series”
- Page 43, Line 1008: *Clarify what you mean by "cycle" in my previous comment in this sentence and this should be more clear.*—See comment above. Inserted “replacement”
- Page 45, Line 1044: *This does not necessarily have to be its own section. Fig. 18 is essentially a summary of what was built upon in the previous section.*—Deleted the heading “Postcanine replacement model”
- Page 45, Line 1046: Replaced “is not perfect for the following reasons” with “has two limitations”
- Page 47, Line 1099: Inserted “within Therapsida”
- Page 47, Line 1106: Replaced “too” with “as well”
- Page 48, Lines 1129–1131: *Definitely an appropriate conclusion, given the data and comparisons. What about the postcanine replacement patterns/frequency?*—Inserted “In contrast, the apparent retention of the first tooth of the postcanine series in *Galesaurus* is considered a basal condition. *Galesaurus* therefore has a suite of dental characters that are concurrently more basal and more derived than those described in *Thrinaxodon*.”
- Fig 18: *Add a label for stages 1–7 indicating that these are hypothetical, just for clarification. This is important, because it's unlikely that tooth initiation started at locus 1 in the PC series. There are plenty of papers discussing tooth initiation patterns in vertebrates, which point to all kinds of ways that a tooth series may initiate.*—See detailed response below

*My one non-organizational comment would be to make sure the authors include virtual CT slices of all of the specimens for which CT data were available. I understand there are good reasons not to include the full CT datasets for this manuscript, but make sure that enough CT slices are included to corroborate all of the interpretations made in this paper. I think most if not all of them are here, but the more the better. Any additional, good-quality CT images of the dentine fragments from the incisors/canines/postcanines would be worthwhile to include either as in-text figures or as supplementary data.*

—We have included several additional figures showing virtual transverse sections through the anterior and postcanine dentitions of RC 845 (Figs 3 and 7) and BP/1/4602 (Figs 5 and 10).

*Aside from that and my minor in-text comments, I thought the paper was well-written, fairly clear and concise, and provided worthwhile interpretations of the data. I recommend a minor change to one of the last figures as well.*

*―*We have added a clarification to Fig 23 (fig 18 of original submission) that Developmental Stages 1–7 represent hypothetical stages not preserved in the currently known specimens of *Galesaurus*.

**Reviewer 2**

**Suggestion 1**

*The several sentences of the abstract (lines 33-41) should be re-arranged slightly so the delivery would be more orderly. I suggest these changes of the abstract*

*How about change to “…The growth series of* Galesaurus *shows that the incisors and canine continue to develop and replace, even in the largest (presumably the oldest) specimens of* Galesaurus*. In* Galesaurus*, replacement of the canine ceased with the attainment of skeletal maturity, whereas in* Thrinaxodon*, replacement of the canines continued into adulthood. In the maxilla, the first postcanine is consistently the smallest tooth, showing a proportional reduction in size as skull length increased. The first postcanine locus underwent longer development and sustained its replacement in* Galesaurus *than in* Thrinaxodon*. A longer retention of this tooth is a key difference of* Galesaurus *from* Thrinaxodon *in which the anterior-most postcanines are lost after replacements. This difference have contributed to the lengthening of the postcanine series as teeth continue to be added to the posterior end of toothrow through ontogeny in* Galesaurus*, which differs from* Thrinaxodon*. Overall, there are considerable differences between* Galesaurus *and* Thrinaxodon *relating to the replacement and development of their teeth.*

―The abstract was modified following the reviewer’s suggestions.

**Suggestion 2**

*The paper will be better if it can add an illustration of CT visualization of the in situ teeth in the mandible in the medial view of the jaw. It is important to illustrate this view, easily obtainable from the CT scans. Several dental replacement features can be shown.*

*In* Thrinaxodon *the dental lamina groove (Crompton’s groove) is present on along the margin of the dental alveoli, in the medial view of the mandible. The authors have clearly explained that the tooth alveoli are not ankylosed in* Galesaurus*, and the gomphodont tooth implant in the alveoli are permanent. By comparison, in* Thrinaxodon *the deciduous tooth become ankylosed in the dentary (alveoli would be ossified) before the tooth was shed and lost. The dental lamina groove is related to this pattern in* Thrinaxodon*. Does* Galesaurus *have the dental lamina groove (for replacement). If dental lamina groove is absent, then there is one more difference from* Thrinaxodon*.*

*I suggest that the authors can add this description/discussion with a figure on* Galesaurus*. This will make the paper better by providing more comparative information to* Thrinaxodon*.*

―The medial view of the right mandible of AMNH FARB 2227 was illustrated by (Pusch *et al.*, 2019: fig. 16B), however the authors did not comment on the presence of a groove to house the dental lamina. The dental lamina groove is visible in the virtual horizontal sections through the postcanine series of adult specimens (Figs 15 and 17 [figs 10 and 12 of first submission]). The label “dl” has been added to the figures, and the figure captions updated to include the abbreviation “dl, dental lamina groove.” Additionally, Rigney (1938) described a canal lingual to the postcanine series, and the following sentences were inserted on P. 42: “Furthermore, Rigney [63] described two canals in the dentary lingual to the postcanine series. The description of these canals is similar to that of the longitudinal grooves described in the maxilla and dentary of *Thrinaxodon* [40]. Crompton [40] proposed that these grooves housed the dental lamina. Similar lingual grooves extending the length of the postcanine series are evident in two adult specimens of *Galesaurus* (BP/1/5064 and SAM-PK-K10468, Figs 15 and 17).”

**Suggestion 3**

*There are now several CT scan studies on* Galesaurus *skulls (Jasinoski and Abdala 2017; Pusch et al. 2019, and the present manuscript by Norton et al.). But these otherwise excellent papers have not shown a good CT visualization of the postcanine tooth crown. Tooth crowns of* Galesaurus *are the most unique, among cynodonts , and are quite different from those of* Thrinaxodon*. Can you illustrated an upper PC crown in medial view, and lateral view. And then do the same for a lower postcanine? The line drawings in Figure 1 are just to simple, and do not show the lingual views.*

*This will make the paper much more useful, for other paleontologists.*

―A new figure (Fig 6) showing the labial and lingual crown morphology of two upper and two lower postcanine teeth was added.

**Suggestion 4 (also a neutral comment)**

*The upper canine can be open-rooted, even in larger and presumably the older skulls (e.g., NMQR135; BP/1/5064). This is most interesting. This could be due to the fact the canine was still slowly growing, or that the tooth is high-crown, or deep rooted, or both.*

*Interestingly, there is an analogous pattern in the Jurassic haramiyidan* Vilevolodon*. The replacing lower incisor can also be open-rooted, in the incisor locus that experienced slow tooth growth and replacement in some of these haramiyidans (Luo et al. 2017: extended data figures 4 and 6). I think the open-rooted pattern of incisors in the haramiyidan* Vilevolodon *may be convergent in the tooth growth mode to the open-rooted and high-crown canines (but obviously these incisor and canine are in different tooth positions).*

*This is consistent with the authors’ interpretation that* Galesaurus *has a relatively slow, and sustained tooth replacement.*

―Although we agree that the roots of the replacement incisors of *Vilevolodon* are open-rooted, we disagree with comparing them to the maxillary canines of the larger specimens of *Galesaurus* (e.g., BP/1/5064 and NMQR 135). The main reason, in these two *Galesaurus* specimens the open-rooted tooth is already functional, whereas in *Vilevolodon* the open-rooted tooth is clearly a developing replacement tooth with the functional tooth still *in situ*. It would perhaps be better to compare the condition in the mandibular incisors of *Vilevolodon* to those of the maxillary canines in the smaller specimens of *Galesaurus* (e.g., RC 845 and BP/1/4602), or perhaps even better, the left mandibular canine of BP/1/4602. In these examples, the replacing tooth may be considered as open-rooted as they were still in the process of developing, and the corresponding functional teeth have yet to be exfoliated.

**Additional changes**

The *PLOS ONE* guidelines were consulted, and several minor formatting changes were made. These include the spacing and formatting of figure captions, the deletion of unnecessary spaces in table footnotes, etc.

Figure numbering in captions and citations were updated following the addition of the five figures suggested by the reviewers, as well as the rearrangement in the order of citation for existing figures.

In several figure captions, “transverse cross-section” was changed to “horizontal section” to match the terminology used by Abdala et al (2013) and the online database DigiMorph (www.digimorph.org).

In addition to the recommendations made by the reviewers, several additional changes were made to the manuscript. For the sake of completeness, these changes are listed below:

- Page 4, Lines 114–115: Reorganised the wording of the sentence.
- Page 5, Line 124: Updated “10 years” to “20 years”
- Page 6, Line 126: Moved “(AMNH FARB 2227)”.
- Page 10, Line 211: Inserted the label “(1)” for the equation.
- Page 10, Line 212: Changed “original value” to read “actual specimen measurement”
- Page 10, line 213: Inserted “measured”
- Page 12, Lines 249–250 [Fig 4 caption]: Inserted “of the palate”
- Page 13, Line 270: Inserted “tall,”
- Page 15, Line 316: Changed “the left” to read “both maxillae”
- Page 15, Lines 320–321: Deleted “developing in the dentaries”
- Page 15, Lines 328–331: Reorganised and expanded upon description for clarity.
- Page 15, Lines 333–334: Deleted the sentence “An old remnant root between PC6 and PC7 is preserved labially (not visible in medial view).”
- Page 17, Line 379: Changed “five” to “four” to match updated interpretation of the distal-most tooth in the series.
- Page 17, Line 382: Inserted “PC” for consistency with formatting in rest of manuscript
- Page 18, Lines 388–389: Reorganised the wording of the sentence
- Page 21, Lines 466–467: Deleted “either prior to, or during fossilization”
- Page 22, Line 486: Reorganised wording of the sentence.
- Page 26, Lines 580–581: Deleted the sentence “The germ lingual to pc6 is of similar size to the mineralized crown of the replacement pc12.”
- Page 30, Line 687: Changed “an” to “and”
- Page 31, Line 705: Inserted “preserved”
- Page 33, Lines 760–761: Reorganised word order.
- Page 36, Line 829: Inserted “of *Thrinaxodon*”
- Pages 39–40, Lines 917–919: Moved “dugongs [97]” and pluralised narwhal and hippopotamus for consistency.
- Page 40, lines 932–933: Deleted sentence “This implies that individuals may also have attained sexual maturity.”
- Page 44, Line 1019: Changed “anterior” to “mesial”
- Page 44, Line 1020: Inserted “apparently”
- Page 44, Lines 1022–1023: Inserted the sentence “However, this is based on the assumption that the mesial and distal teeth belong to the same replacement wave.”
- Pages 47–48, Lines 1116–1120: Inserted the sentences “Furthermore, Abdala et al. [19] suggested that due to the lack of occlusion between opposing postcanines, it is likely that *Galesaurus* did not process food in the oral cavity. Instead, the postcanines may have been used to manipulate prey such that it could be swallowed whole, in a manner comparable to that observed in most extant carnivorous lizards [114].”
